# Supplementary material for: LSSmScarlet, dCyRFP2s, dCyOFP2s and CRISPRed2s, Genetically Encoded Red Fluorescent Proteins with a Large Stokes Shift
Source: Int J Mol Sci. 2021 Nov 28;22(23):12887. doi: 10.3390/ijms222312887 (PMC8657457; doi:10.3390/ijms222312887)
Supplement: Supplementary file 1 [file ijms-22-12887-s001.zip › ijms-1393183-supplementary.pdf]

Supplementary Information

LSSmScarlet, dCyRFP2s, dCyOFP2s and CRISPRed2s, Genetically Encoded Red Fluorescent Proteins with a Large Stokes Shift

Oksana M. Subach, Anna V. Vlaskina, Yuliya K. Agapova, Pavel V. Dorovatovskii, Alena Y. Nikolaeva, Olga I. Ivashkina, Vladimir O. Popov, Kiryl D. Piatkevich, Maria G. Khrenova, Tatiana A. Smirnova, Konstantin M. Boyko, and Fedor V. Subach

Supplementary Tables and Figures

**Table S1**	**2**
**Table S2**	**3**
**Table S3**	**4**
**Figure S1**	**6**
**Figure S2**	**7**
**Figure S3**	**8**
**Figure S4**	**9**
**Figure S5**	**10**
**Figure S6**	**11**
**Figure S7**	**12**
**Figure S8**	**13**
**Figure S9**	**14**
**Supplementary references**	**15**

Table S1. Molecular brightness of the available and developed LSSRFPs.

LSSRFP protein	Brightness vs EGFP (%)	Brightness at 488nm exc (%)	Reference
**CyOFP1**	102 (90)	97 (87)	this paper ([1])
**CyRFP1**	109	81	[2]
**dCyOFP2s**	73	73	this paper
**dCyRFP2s**	73	65	this paper
**mCyRFP1**	55 (52)	49 (44)	this paper ([2])
**mCRISPRed**	39	34	[3]
**LSSmScarlet**	39	34	this paper
**mBeRFP**	52	25	[4]
**CRISPRed2s**	32	24	this paper
**LSSmCherry1**	30	20	[5]
**LSSmOrange**	70	9	[6]
**mKeima**	10	3	[7]

Table S2. Maturation efficiency of the LSSRFPs.

Proteins	ε^{280} ($mM^{-1}\cdot cm^{-1}$) [a]	ε^{max} ($mM^{-1}\cdot cm^{-1}$) [b]	Folding efficiency (%) [c]
**LSSmScarlet**	27.4	30.2 ± 0.6	91
**mCyRFP1**	30.5	30.6 ± 0.5	100
**dCyRFP2s**	32.0	42 ± 2	76
**CyOFP1**	30.5	45.1 ± 0.2	68
**dCyOFP2s**	30.5	36 ± 4	85
**mCRISPRed**	27.5	29.1 ± 0.9	95
**CRISPRed2s**	29.0	28.7 ± 0.9	101

[a] Extinction coefficients at 280 nm were calculated using ProtParam tool (https://web.expasy.org/protparam/). [b] Extinction coefficients were determined by alkaline denaturation. Standard deviations are shown. [c] Maturation efficiency was calculated as $\varepsilon^{280}/\varepsilon^{max}$*100%.

Table S3. List of primers.

Primer	Primer sequence (5′-3′)	Comment
mSc-BglII	gacAGATCTATGGTGAGCAAGGGCGAG	Use for LSSmScarlet
mCherry-EcoRI-r	tcgaattcttacttgtacagctcgtccatg	
Fw-BglII-(PA)TagRFP	GCTCGAGATCTATGGTGTCTAAGGGCGAAGAG	Use for mCRISPRed
Rv-LSSmOrange-EcoRI	CAGGAATTCTTACTTGTACAGCTCGTCC	Use for mCRISPRed and mCyRFP
Fw-LSSmOrange-BglII	gacAGATCTATGGTGAGCAAGGGCGAGGAG	Use for mCyRFP1
mSc-148DE	GACAATGGGCTGGGAAGCGGAWACCGAGCGGTTGTACCCC	Overlap library 1
mSc-148DE-r	GGGGTACAACCGCTCGGTWTCCGCTTCCCAGCCCATTGTC	
mSc-165,7-X	GACGGCGTGCTGAAGGGCGACNNSAAGNNSGCCCTGCGCCTGAAGGACGG	
mSc-165,7-X-r	CCGTCCTTCAGGCGCAGGGCSNNCTTSNNGTCGCCCTTCAGCACGCCGTC	
mSc-148X	GACAATGGGCTGGGAAGCGNNSACCGAGCGGTTGTACCCC	Overlap library 2 and 3
mSc-148X-r	GGGGTACAACCGCTCGGTSNNCGCTTCCCAGCCCATTGTC	
mSc-165,7-DX	GACGGCGTGCTGAAGGGCGACGAWAAGNNSGCCCTGCGCCTGAAGGACGG	Overlap library 2
mSc-165,7-DX-r	CCGTCCTTCAGGCGCAGGGCSNNCTTWTCGTCGCCCTTCAGCACGCCGTC	
mSc-165,7-XD	GACGGCGTGCTGAAGGGCGACNNSAAGGAWGCCCTGCGCCTGAAGGACGG	Overlap library 3
m Sc-165,7-XD-r	CCGTCCTTCAGGCGCAGGGCWTCCTTSNNGTCGCCCTTCAGCACGCCGTC	
mSc-EcoRI-r2	tcgaattccttgtacagctcgtccatg;	Use for LSSmScarlet; no stop codon to clone to sfGFP
LSSCy-KpnI	tccggtaccgccaccATGGTGAGCAAGGGCGAG	Use for LSSmScarlet, CyRFPs, CyOFPs
LSSCR-KpnI	tccggtaccgccaccATGGTGTCTAAGGGCGAAG	
LSSCy-AgeI-r	tgaaccggtcgCTTGTACAGCTCGTCCATG	Use for LSSmScarlet, mCyRFP1, dCyRFP2s, CyOFP1, dCyOFP2s
LSSCR-AgeI-r	tgaaccggtcgCTTGTACAGCTCGTCCATC	Use for mCRISPRed and CRISPRed2s
NheI-LSSmSc	tccgctagcggtcgccaccATGGTGAGCAAGGGCGAG	LSSmScarlet into pTagBFP-actin at NheI/HindIII, pTagGFP2-tubulin at NheI/BsrGI sites
LSSmSc-HindIII-	attaagcttGTACAGCTCGTCCATG	

**r**		
**mSc-BglII**	gacAGATCTATGGTGAGCAAGGGCGAG	LSSmScarlet into pAAV-AscI-CAG-H2B-B-GECO1 at BglII/HindIII sites
**mCherry-HindIII-r**	GAT AAG CTT TTA CTT ATA CAG CTC GTC	
**LSSmSc-XhoI**	agcctcgagATGGTGAGCAAGGGCGAG	LSSmScarlet into pAAV-CAG-dMito-mCherry at XhoI/EcoRI sites (dMito = MSVLTPLLLRGLTGSARRLPVPRAKIHSLGPARSSVLTPLLLRGLTGSARRLPVPRAKIHSLGPARSAS)
**mCherry-EcoRI-r**	tcgaattcttacttgtacagctcgtccatg	
**LSSmSc-BamHI**	ccgggatccaccggtcgccaccATGGTGAGCAAGGGCGAG	LSSmScarlet into pLU-CMV-vimentin-NeonOxIrr at BamHI/BsrGI sites
**LSSmSc-XbaI-r**	tgatctagattaCTTGTACAGCTCGTCCATG	
**LSSmSc-198I**	GGCGCCTACAACGTCGACATCAAGTTGGACATCACCTCC	Mutagenesis at position 198.
**LSSmSc-198I-r**	GGAGGTGATGTCCAACTTGATGTCGACGTTGTAGGCGCC	
**LSSmSc-198Y**	GGCGCCTACAACGTCGACTACAAGTTGGACATCACCTCC	
**LSSmSc-198Y-r**	GGAGGTGATGTCCAACTTGTAGTCGACGTTGTAGGCGCC	
**LSSmSc-198K**	GGCGCCTACAACGTCGACaagAAGTTGGACATCACCTCC	
**LSSmSc-198K-r**	GGAGGTGATGTCCAACTTcttGTCGACGTTGTAGGCGCC	
**LSSmSc-198H**	GGCGCCTACAACGTCGACCaCAAGTTGGACATCACCTCC	
**LSSmSc-198H-r**	GGAGGTGATGTCCAACTTGtGGTCGACGTTGTAGGCGCC	
**LSSmSc-198E**	GGCGCCTACAACGTCGACgagAAGTTGGACATCACCTCC	
**LSSmSc-198E-r**	GGAGGTGATGTCCAACTTctcGTCGACGTTGTAGGCGCC	

LSSmScarlet-stop gene:

ATGGTGAGCAAGGGCGAGGCAGTGATCAAGGAGTTCATGCGGTTCAAGGTGCACATGGAGGGCTCCATGAACGGCCACGAGTTC
GAGATCGAGGGCGAGGGCGAGGGCCGCCCCTACGAGGGCACCCAGACCGCCAAGCTGAAGGTGACCAAGGGTGGCCCCCTGCCC
TTCTCCTGGGACATCCTGTCCCCTCAGTTCATGTACGGCTCCAGGGCCTTCATCAAGCACCCCGCCGACATCCCCGACTACCATAA
GCAGTCCTTCCCCGAGGGCTTCAAGTGGGAGCGCGTGATGAACTTCGAGGACGGCGGCGCCGTGACCGTGACCCAGGACACCTCC
CTGGAGGACGGCACCCTGATCTACGAGGTGAAGCTCCGCGGCACCAACTTCCCTCCTGACGGCCCCGTAATGCAGAAGAAGACA
ATGGGTTTGGAAGCGGACACCGAGCGGTTGTACCCCGAGGACGGCGTGCTGAAGGGCGACATTAAGATGGCCCTGCGCCTGAAG
GACGGCGGCCGCTACCTGGCGGACGTCAGGACCACCTACAAGGCCAAGAAGCCCGTGCAGATGCCCGGCGCCTACAACGTCGAC
CGCAAGTTGGACATCACCTCCCACAACGAGGACTACACCGTGGTGGAACAGTACGAACGCTCCGAGGGCCGCCACTCCACCGGC
GGCATGGACGAGCTGTACAAGTAA

dCyRFP2s-stop gene:

ATGGTGAGCAAGGGCGAGGAGCTGATCAAGGAGTCCATGAGAAGCAAGCTGTACCTGGAAGGTAGCGTGAACGGCCACC
AGTTCAAGTGCATCCACGAAGGGGAGGGCAAGCCCTACGAGGGCAAGCAGACCGCGAGGATCAAGGTGGTGGAGGGGG
GCCCCCTGCCGTTCGCATTCGACATCCTGGCCACCATGTTTATGTACGGGAGCAAGGTGTTCATCAAGTACCCCGCCGACCT
CCCCGATTATTTTAAGCAGTCCTTCCCTGAGGGCTTCACATGGGAGAGAGTCATGGTGTTCGAAGACGGGGGCGTGCTGAC
CGCCACTCAGGACACCAGCCTCCAGGACGGCGGGCTCATCTACAACGTCAAGCTCAGAGGGGTGAACTTCCCAGCCTACG
GCCCCGTGATGCAGAAGAAAACACTGGGCTGGGAGCCCAGCACCGAGACCATGTACCCCGCTGACGGCGGCCTGGAAGG
CAGGTGCGACAAGTTGCTGAAGCTCGTGGGCGGGGGCCACCTGCACGTCAACTTCAAGACCACATACAGGTCCAAGAAAC
CCGTGAAGATGCCCGGCGTCCACTACGTGGACCGCAGACTGGAAAGAATCAAGGAGGCCGACAACGAGACCTACGTCGA
GCAGTACGAGCACGCTGTGGCCAGATACTCCAACCTGGGCGGAGGCATGGACGAGCTGTACAAGTAA

dCyOFP2s-stop gene:

ATGGTGAGCAAGGGCGAGGAGCTGATCAAGGAGAACATGAGAAGCAAGCTGTACCTGGAAGGCAGCGTGAACGGCCACC
AGTTCAAGTGCACCCACGAAGGGGAGGGCAAGCCCTACGAGGGCAAGCAGACCAACAGGATCAAGGTGGTGGAGGGAG
GCCCCCTGCCGTTCGCATTCGACATCCTGGCCACCCACTTTATGTACGGGAGCAAGGTGTTCATCAAGTACCCCGCCGACCT
CCCCGATTATTTTAAGCAGTCCTTCCCTGAGGGCTTCACATGGGAGAGAGTCATGGTGTTCGAAGACGGGGGCGTGCTGAC
CGCCACCCAGGACACCAGCCTCCAGGACGGCGAGCTCATCTACAACGTCAAGGTCAGAGGGGTGAACTTCCCAGCCAACG
GCCCCGTGATGCAGAAGAAAACACTGGGCTGGGAGCCCAGCACCGAGACCATGTACCCCGCTGACGGCGGCCTGGAAGG
CAGATGCGACAAGGCCCTGAAGCTCGTGGGCGGGGGCCACCTGCACGTCAGCTTCAAGACCACATACAAGTCCAAGAAA
CCCGTGAAGATGCCCGGCGTCCACTACGTGGACCGCAGACTGGAAAGAATCAAGGAGGCCGACAACGAGACCTACGTCG
AGCAGTACGAGCACGCTGTGGCCAGATACTCCAACCTgGGCGGAGGCATGGACGAGCTGTACAGGTAA

CRISPRed2s-stop gene:

ATGGTGTCTAAGGGCGAAGAGCTGATCAAGGAATATATGCGTATGAAGGTGGTCATGGAAGGTTCGGTCAACGGCCACCA
ATTCAAATGCACAGGCGAAGGAGAAGGCCGACCGTACGAAGGAGTGCAAACCATGAGGATTAAAGTCATCGAGGGAGGA
CCCCTGCCATTTGCCTTTGACATTCTTGCCACGTCGTTCATGTATGGCAGCCGTACTTTTATCAAGTACCCGGCCGATATTCC
TGATTTCTTTAAACAGTCCTTCCCTGAGGGTTTTACTTGGGAAAGAGTTACGAGATACGAAGATGGTGGAGTCGTCACCGTC
ACCCAGGACACCAGCCTTGAGGATGGCGAACTCGTTTACAACGTCAAAGTCAGTGGGGTAAACTTTCCCTCCAATGGTCCC
GTGATGCAGAAGAAGACCAAGGGCTGGGAGGCCGACACAGAGATGATGTATCCTGCAGATGGTGGTCTGAGGGGATACC
TTGATCGGGCACTGAAAGTTGATGGTGGTGGCCATCTGCATTGCAACTTCGTAACAACTTACAGGTCAAAAAAGACCGTCG
GGGACATCAAGATGCCCGGTGTTCATGCCGTTGATCACCGGCTGGAAAGGATCGAGGAAAGTGACAATGAAACCTACGTA
GTACAACGCGAAGTCGCAGTTGCCAAGTACAGCGACCTTGGTGGTGGGATGGACGAGCTGTACAAGTAA

Figure S1. Nucleotide sequences of the LSSmScarlet, dCyRFP2s, dCyOFP2s and CRISPRed2s proteins.

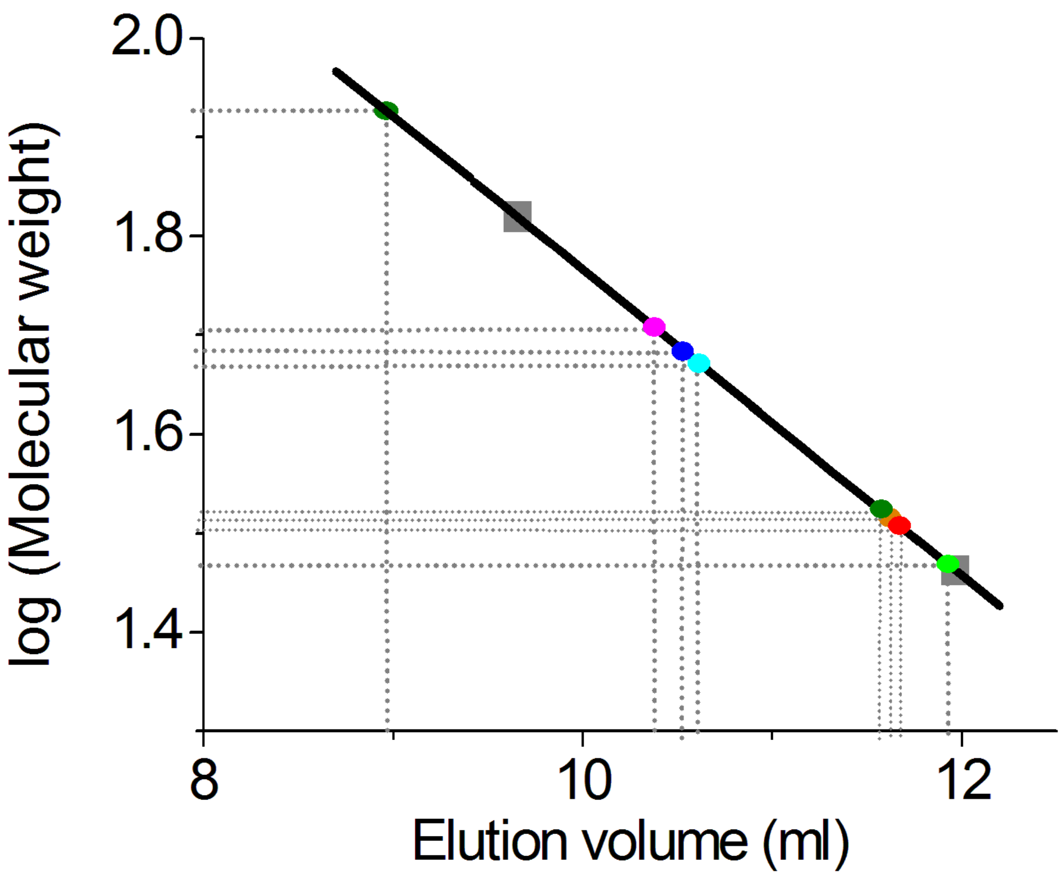

Figure S2. Calibration curve for **f**ast protein liquid chromatography of LSSRFPs proteins. LSSRFPs were eluted in 20 mM Tris-HCl (pH 7.80) and 200 mM NaCl buffer. The molecular weights of LSSRFPs were calculated from a linear regression of the dependence of logarithm of control molecular weights vs elution volume as depicted on Figure 2c. Controls of molecular weights of 66 and 29 kDa, LSSmScarlet, mCyRFP1, dCyRFP2s, CyOFP1, dCyOFP2s, mCRISPRed, and CRISPRed2s are shown in grey, red, green, blue, cyan, magenta, orange, and olive colors, respectively.

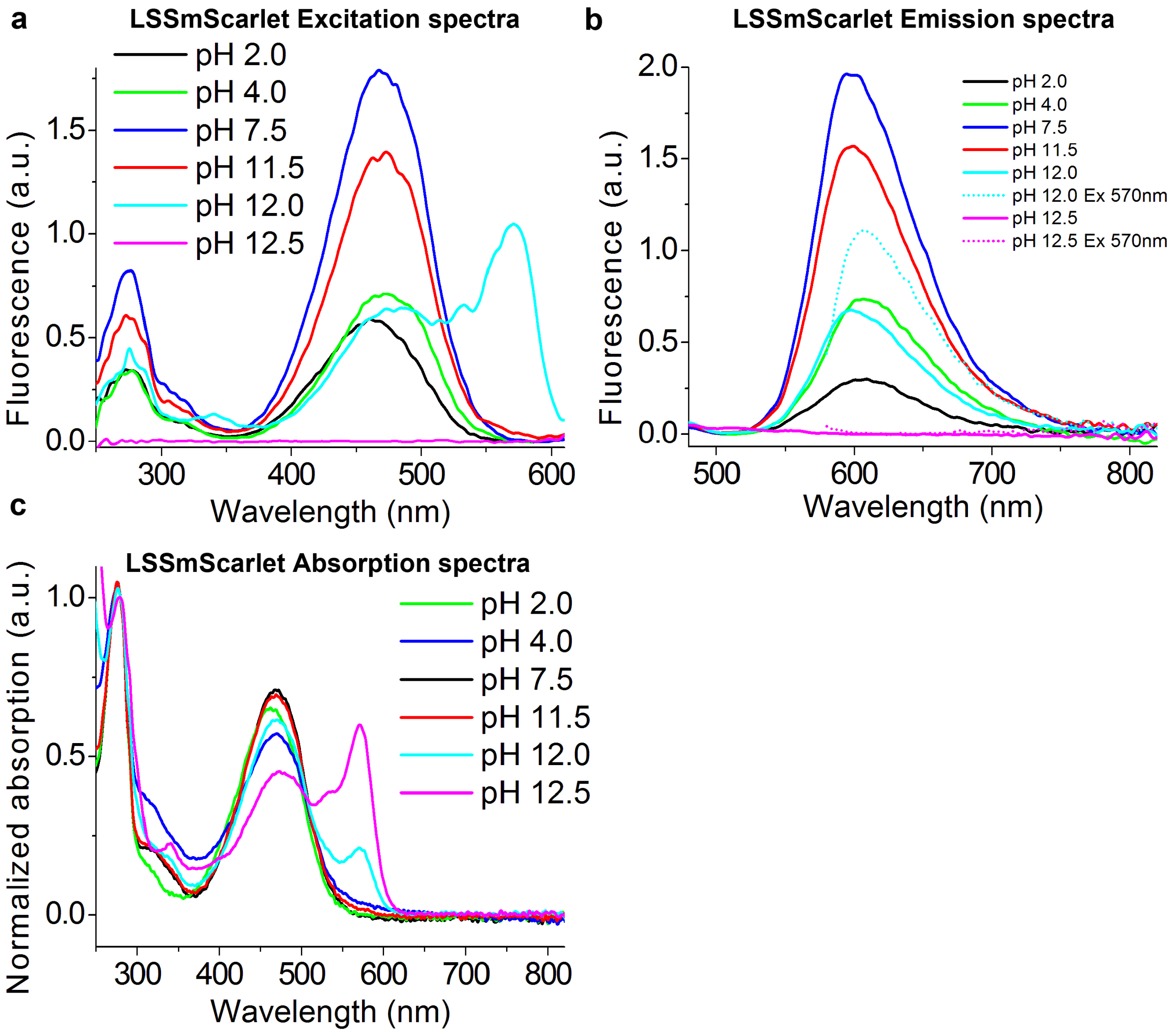

Figure S3. Excitation, emission and absorption spectra for the LSSmScarlet protein at different pH. (**a, b**) Spectra for purified LSSmScarlet protein (45 nM final concentration) were measured in buffers 30 mM citric acid, 30 mM borax, 30 mM NaCl with pH ranging from 2.0 to 12.5, at room temperature, using a CM2203 spectrofluorometer (Solar, Minsk, Belarus). (**c**) Absorption spectra for purified LSSmScarlet protein (69 μM final concentration) were measured in buffers 30 mM citric acid, 30 mM borax, 30 mM NaCl with pH ranging from 2.0 to 12.5, at room temperature, using a NanoDrop 2000c Spectrophotometer (Thermo Scientific, DE, USA).

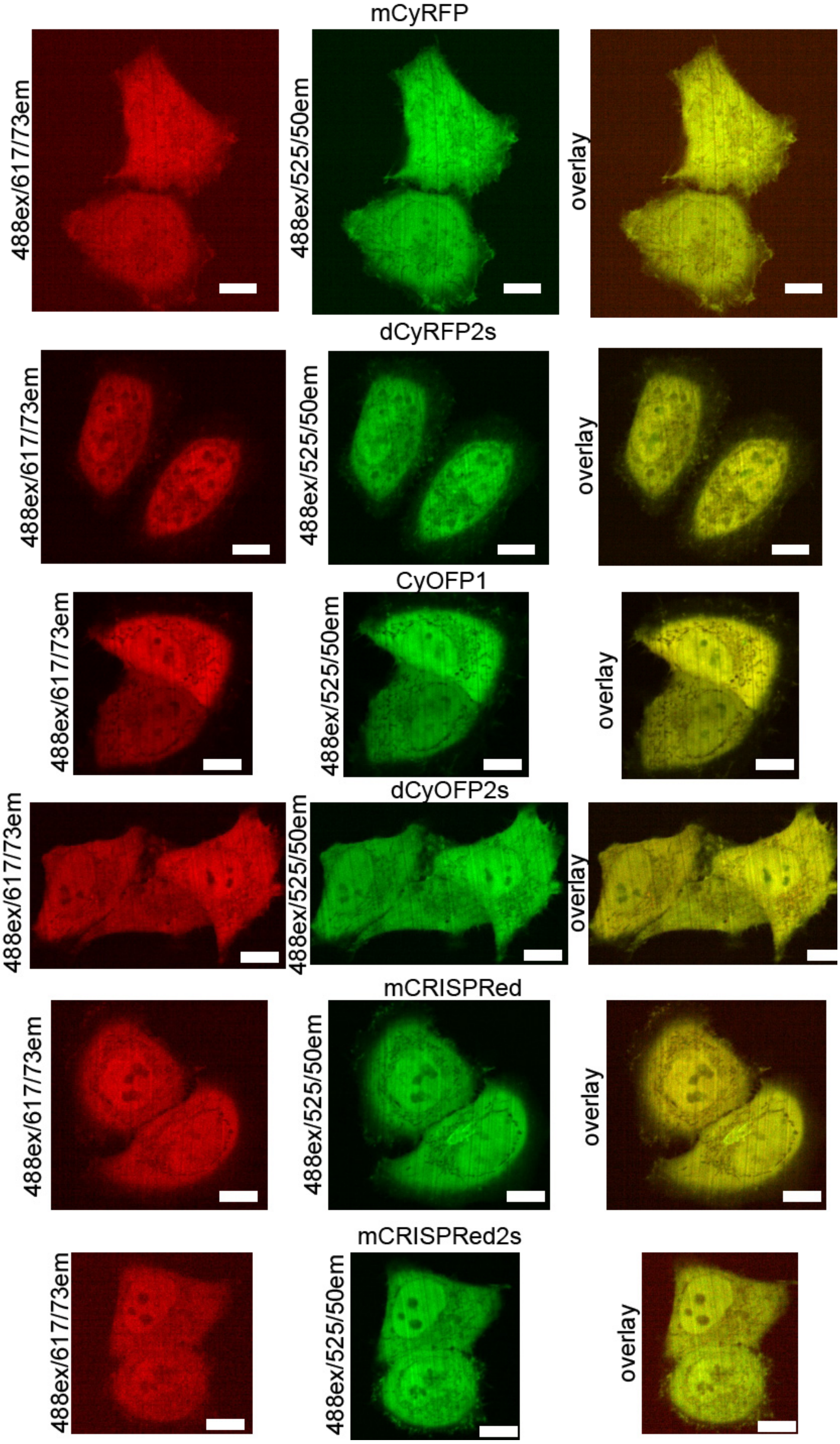

Figure S4. Localization of the LSSRFPs-P2A-EGFP fusions expressing in mammalian cells. Confocal images of HeLa cells expressing the mCyRFP1-P2A-EGFP, dCyRFP2s-P2A-EGFP, CyOFP1-P2A-EGFP, dCyOFP2s-P2A-EGFP, mCRISPRed-P2A-EGFP, and CRISPRed2s-P2A-EGFP fusion proteins. The EGFP protein is connected to LSSRFPs via P2A-self cleavable linker. Scale bar, 100 μm.

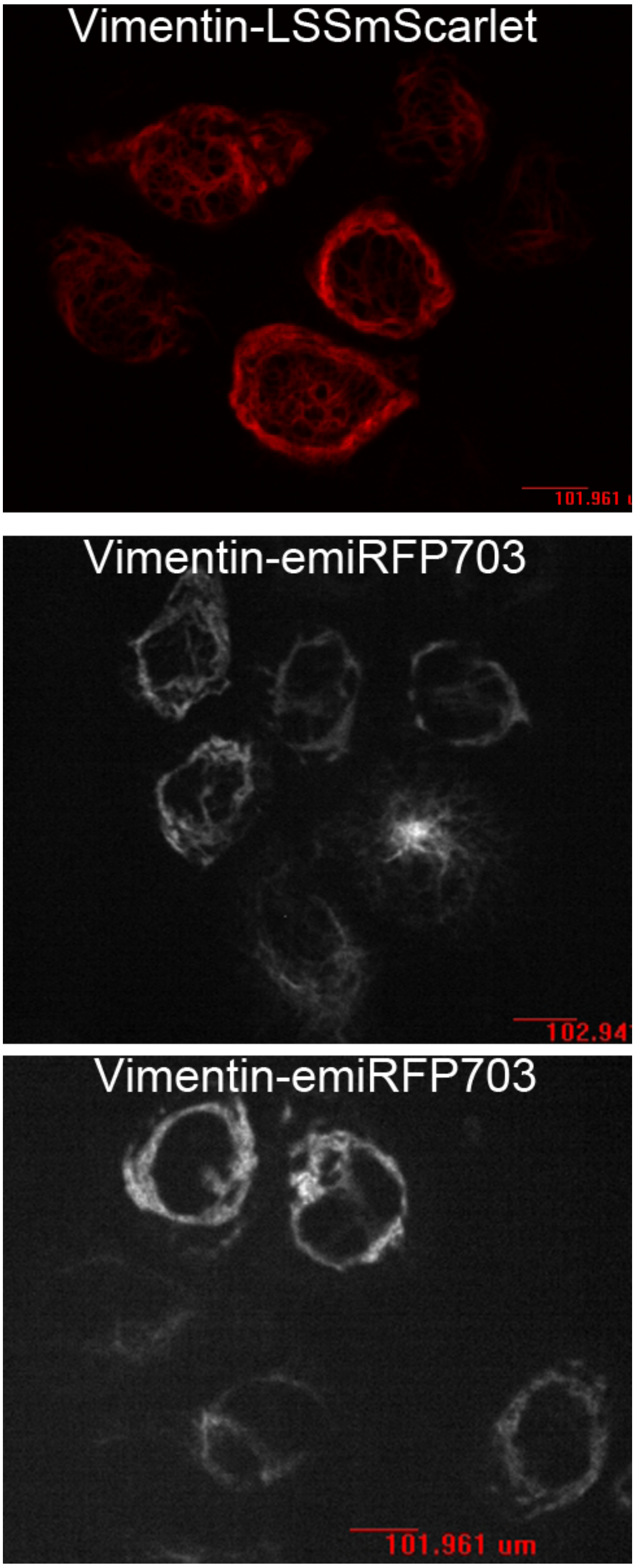

Figure S5. Comparison of localization of the LSSmScarlet and emiRFP703 proteins in fusion with vimentin in mammalian cells. Confocal images of HeLa cells expressing the vimentin-LSSmScarlet and vimentin-emiRFP703 fusions. Scale bar, 100 μm.

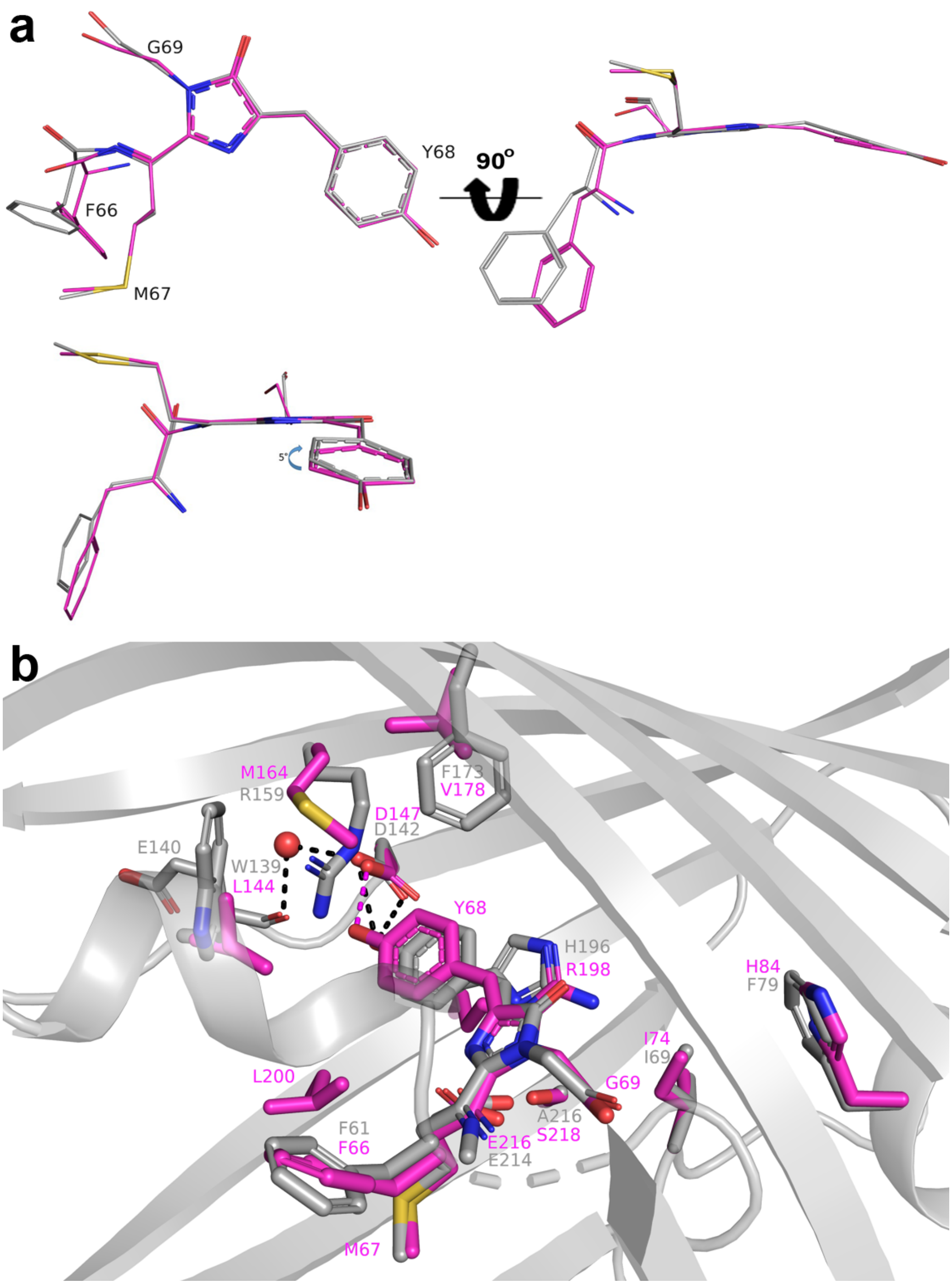

Figure S6. Structural comparison of the chromophores and their immediate environments for the LSSmScarlet and mCRISPRed (PDB: 6xwy) proteins. (**a**) The chromophores are superimposed by all their atoms with corresponding RMSD of 0.17Å, which revealed a small difference in orientation of the tyrosine side chain (about 5° rotation around the CB2-CG2 axis as shown by the blue arrow at the bottom of panel a). The orientation of the chromophores on the right is rotated 90° around the horizontal axis with respect to that on the left. (**b**) Superposition the LSSmScarlet and mCRISPRed by Cα-atoms. D147 of LSSmScarlet makes direct hydrogen bond (magenta dashed line) with chromophore (Y68). D142 of mCRISPRed makes direct hydrogen bond (black dashed line) with chromophore and water mediated hydrogen bond with E140 residue. Residue labels are shown for the LSSmScarlet and mCRISPRed proteins in magenta and gray, accordingly.

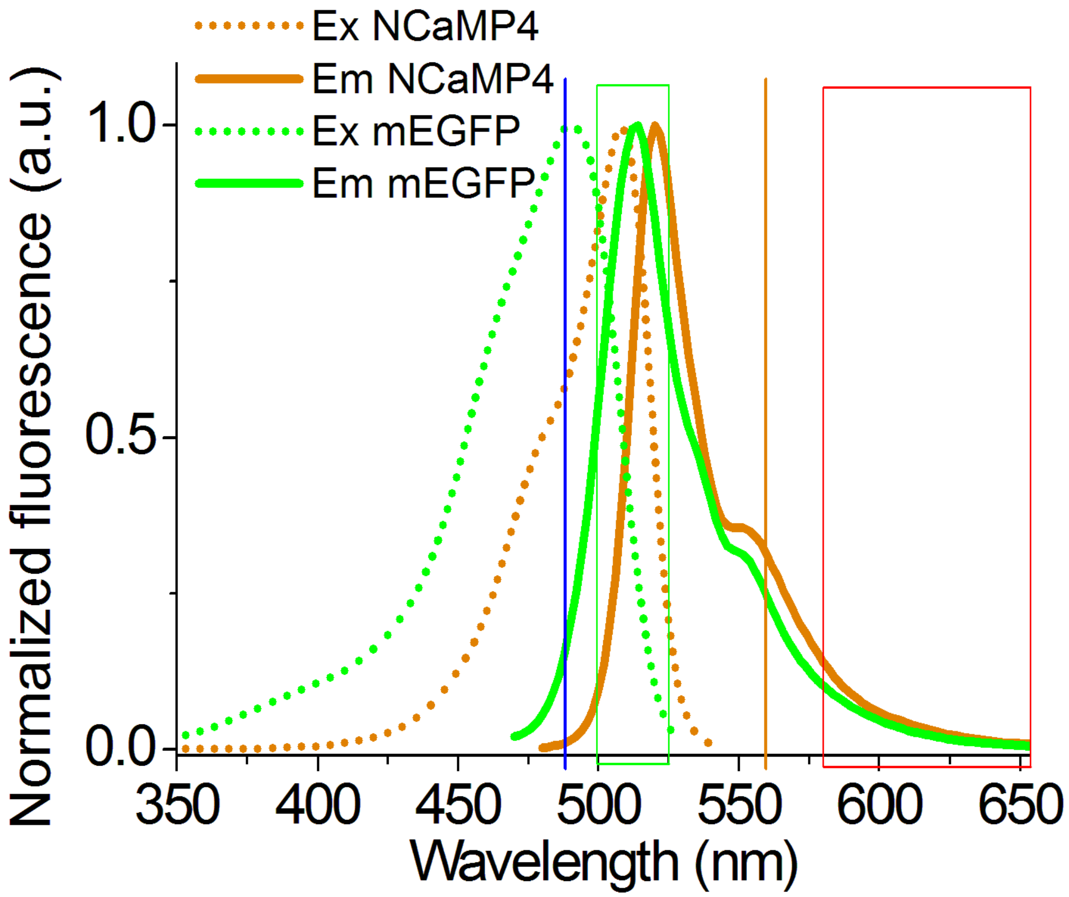

Figure S7. Excitation and emission spectra for the mEGFP protein and the NCaMP4 calcium indicator. 488 and 561 nm excitation laser lines and green/red emission filters used for dual-color imaging are indicated as a blue and orange vertical lines and green/red boxes, respectively.

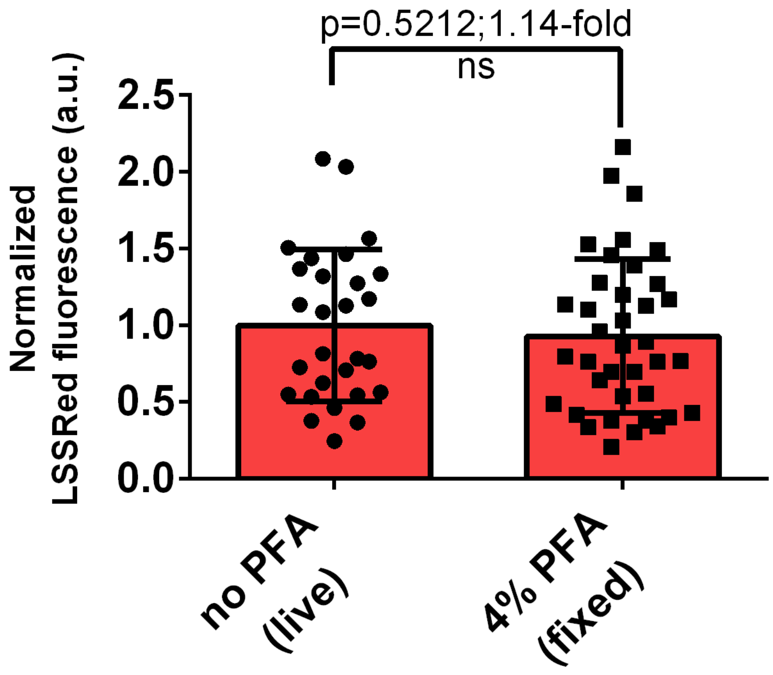

Figure S8. Impact of 4% PFA fixation on the brightness of the H2B-LSSmScarlet protein expressed in HeLa cells. Fixation of the cells was performed with 4% PFA for 15 min at room temperature.

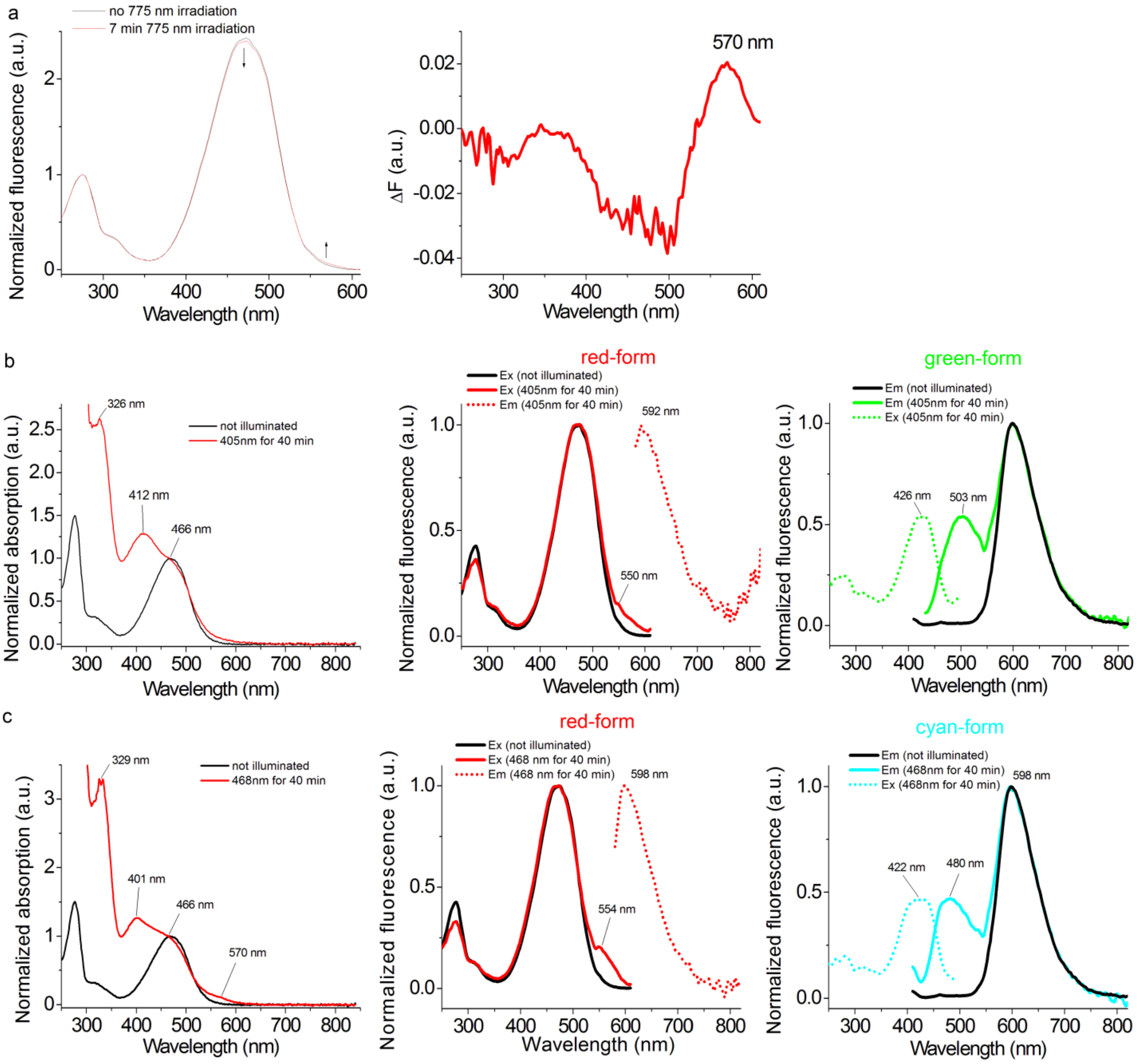

Figure S9. Impact of the illumination on the spectral properties of the purified LSSmScarlet protein. **(a)** (On the left) Excitation spectra for the LSSmScarlet protein before and after 7 min of 2P illumination using 775 nm laser (4010 mW power) through 25x oil objective lens. Excitation spectra were normalized to peak at 280 nm. (On the right) The difference excitation spectrum is shown. **(b)** (On the left) Absorption spectra for the LSSmScarlet protein before and after 40 min of the 405 nm illumination using LED array (57 mW/cm^2 power). Excitation and emission spectra for the traces of the red- (in the middle) and major green-form (on the right) for the illuminated LSSmScarlet protein in comparison with not illuminated one. **(c)** (On the left) Absorption spectra for the LSSmScarlet protein before and after 40 min of the 468 nm illumination using LED array (200 mW/cm^2 power). Excitation and emission spectra for the traces of the red- (in the middle) and major cyan-form (on the right) for the illuminated LSSmScarlet protein in comparison with not illuminated one. Absorption spectra were normalized to peak at 466 nm.

Supplementary references

1. Chu, J.; Oh, Y.; Sens, A.; Ataie, N.; Dana, H.; Macklin, J. J.; Laviv, T.; Welf, E. S.; Dean, K. M.; Zhang, F.; Kim, B. B.; Tang, C. T.; Hu, M.; Baird, M. A.; Davidson, M. W.; Kay, M. A.; Fiolka, R.; Yasuda, R.; Kim, D. S.; Ng, H. L.; Lin, M. Z., A bright cyan-excitable orange fluorescent protein facilitates dual-emission microscopy and enhances bioluminescence imaging in vivo. *Nature biotechnology* **2016,** 34, (7), 760-7.
2. Laviv, T.; Kim, B. B.; Chu, J.; Lam, A. J.; Lin, M. Z.; Yasuda, R., Simultaneous dual-color fluorescence lifetime imaging with novel red-shifted fluorescent proteins. *Nature methods* **2016,** 13, (12), 989-992.
3. Erdogan, M.; Fabritius, A.; Basquin, J.; Griesbeck, O., Targeted In Situ Protein Diversification and Intra-organelle Validation in Mammalian Cells. *Cell Chem Biol* **2020,** 27, (5), 610-621 e5.
4. Yang, J.; Wang, L.; Yang, F.; Luo, H.; Xu, L.; Lu, J.; Zeng, S.; Zhang, Z., mBeRFP, an improved large stokes shift red fluorescent protein. *PloS one* **2013,** 8, (6), e64849.
5. Shen, Y.; Chen, Y.; Wu, J.; Shaner, N. C.; Campbell, R. E., Engineering of mCherry variants with long Stokes shift, red-shifted fluorescence, and low cytotoxicity. *PloS one* **2017,** 12, (2), e0171257.
6. Shcherbakova, D. M.; Hink, M. A.; Joosen, L.; Gadella, T. W.; Verkhusha, V. V., An orange fluorescent protein with a large Stokes shift for single-excitation multicolor FCCS and FRET imaging. *Journal of the American Chemical Society* **2012,** 134, (18), 7913-23.
7. Kogure, T.; Karasawa, S.; Araki, T.; Saito, K.; Kinjo, M.; Miyawaki, A., A fluorescent variant of a protein from the stony coral Montipora facilitates dual-color single-laser fluorescence cross-correlation spectroscopy. *Nat. Biotechnol.* **2006,** 24, (5), 577-81.
